# Editorial: New insights in leprosy (Hansen's disease)

**DOI:** 10.3389/fmed.2024.1372149

**Published:** 2024-02-13

**Authors:** Sebastian Vernal, Ciro Martins Gomes

**Affiliations:** ^1^Sustentabilidade e Responsabilidade Social, Hospital Alemão Oswaldo Cruz, São Paulo, Brazil; ^2^Programa de Pós-Graduação em Ciências Médicas, Universidade de Brasília, Brasília, Brazil

**Keywords:** infectious diseases, neglected tropical disease (NTD), leprosy, mycobacterium, *Mycobacterium leprae*

Leprosy, also known as Hansen's disease, has a long and complex history dating back to ancient times. Traditionally, it has been viewed through fear and isolation, perpetuating social stigmas that have endured for centuries. The quest to understand and combat leprosy is ongoing, with significant advancements in the 20th century. The development of multidrug therapy has proven effective in treating and critically reducing the disease burden worldwide (1). Recently, the scientific community has witnessed a resurgence in interest in leprosy, leading to a cascade of discoveries. This editorial delves into recent strides in leprosy research, shedding light on novel perspectives and potential solutions.

Leprosy transmission has long been scrutinized and recent studies have provided a more nuanced understanding of how the mycobacterium spreads. Studies exploring environmental reservoirs in leprosy-endemic regions have detected *Mycobacterium leprae* DNA in soil and water samples, and animals (ex. armadillos). This finding challenges the conventional belief that the bacterium resides exclusively in humans and prompts a reconsideration of the factors contributing to leprosy transmission (2). Information regarding the role of ticks in the transmission of leprosy is scarce. An interesting study by Krausser et al. weakened the hypothesis that ticks may be involved in leprosy transmission, sustained by the lack of *M. leprae* DNA found in ticks from Eastern Africa. Despite these findings, further studies are required to clarify the roles and interactions between vectors and *M. leprae*.

Accurate and early diagnosis is crucial for effective management of leprosy (3). Our Research Topic includes two outstanding papers that used specialized active searching in the Brazilian Amazon. Costa et al. investigated the occurrence of leprosy among children residing on Caratateua Island and found a high number of new cases in the pediatric population compared to the local baseline records. Bouth et al. focused on the unique genetic characteristics of the identified strain and its implications for drug resistance in leprosy cases. These findings contribute to our understanding of the genetic diversity of *M. leprae* in this region and provide insights into the challenges posed by drug resistance in leprosy control. By shedding light on the specific challenges faced in these areas and the evidence of a high hidden prevalence, both studies underscore the urgency of implementing targeted interventions and healthcare infrastructure, including complementary laboratory tests, to effectively combat and mitigate the spread of Hansen's disease.

Recent innovations in diagnostic tools have paved the way for precise and timely disease identification. Molecular techniques such as polymerase chain reaction have enhanced the sensitivity and specificity of leprosy diagnosis, enabling healthcare professionals to intervene at earlier stages and prevent disease progression (4). Point-of-care diagnostics have also been a game changer, particularly in resource-limited settings. Portable and rapid diagnostic tests allow healthcare workers to conduct on-the-spot assessments, facilitating quicker treatment initiation, and reducing the burden of leprosy on affected individuals and communities (4). Our Research Topic included two studies that underscored this exciting topic. Pierneef et al. discuss a field-friendly serosurvey conducted in Bihar, India, focusing on anti-phenolic glycolipid (PGL)-I antibodies to monitor *M. leprae* transmission in children. This study aimed to develop an efficient and practical method to assess leprosy transmission in a resource-limited setting. The second study by Lima et al. explored the clinical significance and performance of serological testing, focusing on IgA, IgM, and IgG antibodies against Mce1A. The Mce1A protein is part of the Mammalian cell entry (Mce) operon in *M. leprae*, which is involved in entering the mycobacteria into host cells. Mce1A proteins have been studied for their potential significance in leprosy, including research to understand host-pathogen interactions better and, remarkably, as a potential target for leprosy vaccines. Moreover, antibodies against the Mce1A protein have also been investigated as diagnostic and disease progression markers. The study performed by Lima et al. assessed the utility of these antibodies as biomarkers for detecting Hansen's disease and provided valuable insights into leprosy diagnosis.

The integration of artificial intelligence (AI) and machine learning in leprosy research has immense potential. These technologies can be employed in accurate and rapid diagnosis through image recognition technologies aiding in the early identification of leprosy cases and may assist in personalized treatment plans by analyzing individual patient data and optimizing therapeutic outcomes. Additionally, AI can also assist in analyzing vast datasets, identifying patterns, and predicting disease trajectories (5). In this regard, de Andrade Rodrigues et al. explored the application of an AI probabilistic modeling approach based on Bayesian networks to assess the likelihood of leprosy patients experiencing reactions, providing valuable insights into predicting and addressing potential leprosy complications. These applications highlight the promising role of AI in enhancing the efficiency and precision of leprosy research and management strategies.

In the search for predictors of leprosy progression, we also highlight the work of Bezerra-Santos et al., who examined the potential correlation between sTREM-1 and TNF-α, and the severity or progression of leprosy. The roles of these biomarkers are part of a broader network of immune responses to *M. leprae* infection. Research suggests that sTREM-1 is associated with activating myeloid cells and releasing pro-inflammatory cytokines, contributing to the inflammatory processes observed during Hansen's disease. TNF has also been implicated in leprosy immunopathogenesis, associated with granuloma formation, contributing to tissue damage in cutaneous and nerve lesions. The authors' findings suggest that the levels of these biomarkers are linked to clinical outcomes and are promising markers for monitoring and predicting the disease course.

The study of inflammatory reactions to leprosy presents several challenges. The delicate balance between controlling the infection and preventing excessive nerve inflammation is a critical challenge in managing leprosy-associated neuritis. In cases of severe inflammation, immunomodulatory treatments, such as corticosteroids, may be considered to modulate the immune response and mitigate nerve damage; however, using such treatments requires careful consideration of potential side effects and monitoring by healthcare professionals.

Variability in individual responses to reactions, the lack of standardized diagnostic criteria, and the absence of universally accepted treatment guidelines contribute to the complexity of managing these episodes. Our study presents two striking case reports: (i) the first report of two cases of leprosy-associated neuritis that received corticosteroid injections as part of the treatment, providing a new and promising option for leprosy neuritis (Spitz et al.) and (ii) a case report on the use of cyclophosphamide pulse therapy for treating chronic and refractory erythema nodosum leprosum, providing evidence of persistent and difficult-to-treat cases of type 2 leprosy reaction (Machado et al.). The identification of novel drugs guided by large-scale clinical trials is critical for optimizing treatment strategies.

Contact evaluation, a crucial aspect of leprosy control, traditionally relies on identifying and monitoring individuals in close contact with affected patients. Recent advancements in this field have refined contact tracing and assessment strategies, including molecular diagnostic tools and serological tests, leading to a better ability to identify asymptomatic carriers and individuals with early signs of infection. dos Santos et al. focused on identifying and diagnosing neural complications at an early stage among individuals living near patients with leprosy. This study underscores the significance of timely detection in initiating appropriate interventions and highlights the valuable insights gained from the practices of a reference center in Brazil.

Although these insights have marked significant progress in leprosy research, challenges persist in eradicating this disease. Limited funding, regional disparities in healthcare infrastructure, and the need for international collaboration are formidable hurdles. Addressing these challenges requires concerted efforts from governments, non-governmental organizations, and the scientific community to ensure that the momentum gained in leprosy research translates into tangible benefits for those affected. As reported by Montezuma et al., the evidence available in the field of leprosy, even that proposed by leading world references, has very low certainty. Thus, we reinforce the need for more robust data in the field of leprosy to apply the finest evidence-based care during daily assistance to our patients.

Recent strides in leprosy research signify a turning point in the battle against this age-old disease. From diagnostic innovations to active search interventions, a multifaceted approach to understanding and managing leprosy is beginning to yield promising results. As we stand on the cusp of a new era in leprosy research, we must harness collective knowledge and resources to propel these insights into practical solutions, ultimately paving the way for a world free from the shackles of leprosy.

## Author contributions

SV: Writing—original draft, Writing—review & editing. CG: Writing—original draft, Writing—review & editing.
